# A Novel System to Diagnose Cutaneous Adverse Drug Reactions Employing the Cellscan—Comparison with Histamine Releasing Test and Inf-γ Releasing Test

**DOI:** 10.1080/10446670410001722230

**Published:** 2005-03

**Authors:** Ilan Goldberg, Boris Gilburd, Martine Szyper Kravitz, Shmuel Kivity, Berta Ben Chaim, Tirza Klein, Yael Schiffenbauer, Ela Trubniykovr, Sarah Brenner, Yehuda Shoenfeld

**Affiliations:** 1 Department of Dermatology Tel Aviv-Sourasky Medical Center Tel-Aviv Israel; 2 Department of Medicine B Center for Autoimmune Diseases sheba Tel-Hashomer Israel; 3 The Chest and Allergy Institute Tel Aviv-Sourasky Medical Center Tel-Aviv Israel; 4 Drug hypersensitivity laboratory and Tissue Typing Laboratory Rabin Medical Center Beilinson Campus Tel-Aviv Israel; 5 Incumbent of the Laura Schwatz-Kipp Chair for Research on Autoimmune Diseases Tel Aviv University Tel-Aviv Israel

## Abstract

*Background*: There are several mechanisms to describe allergic drug reactions yet the methods to diagnose them are limited.

*Objective*: To compare several conventional clinical and laboratory methods to diagnose skin reactions to drugs to a new method of diagnosing drug reactions by the CellScan system.

*Methods*: The study entailed 21 patients who were diagnosed as suffering from drug eruptions, and 105 healthy controls with no history of drug allergy. The drugs were classified into two groups according to suspicion of causing drug allergy: high and low. Most of the patients were on more than one drug, leading to 41 patient-drug interactions (assays). Histamine releasing test (HRT), interferon (INF)-γ releasing test and CellScan examination were performed on lymphocytes of the patients and controls.

*Results*: The HRT was interpreted as positive in 9 out of 18 (50%) patients and in 13 out of 35 (37%) assays. Based on the INF-γ releasing test, positive results were observed in 16 out of 21 (76%) patients and in 24 out of 41 (59%) assays. In the CellScan test (CST), positive results were observed in 17 out of 21 (81%) patients and in 29 out of 41 (71%) assays. The rate of identifying the drug for eruption in the high suspicion level drugs was 9 out of 22 (41%) assays in the HRT, 20 out of 24 (83%) assays in the INF-γ releasing test, and 21 out of 24 (87%) studies with the CellScan method. The rate of determining of the drug that caused the eruption in the low suspicion level drugs was 4 out of 13 (31%) in the HRT, 4 out of 17 (24%) assays in the INF-γ releasing test, and 8 out of 17 (47%) analyses in the CST. When examined in the CellScan, 99 out of 105 (94%) controls were interpreted as negative.

*Conclusion*: This preliminary study indicates that the CellScan seems to be an easy and promising method for the detection of drugs responsible for adverse skin reactions. In contrast to the HRT and to the Interferon-γ secretion test, the CellScan method is characterized by its ability to track and monitor the reaction of individual cells. By measuring the kinetic parameters of selected cells before and after adding the suspected drug, we were able to identify the culprit drug. The CellScan method had the highest sensitivity, and the interferon-γ secretion test had the highest specificity for detection of the culprit drug. In contrast, the analysis of 105 normal control sera disclosed a high specificity of 94% for the CellScan method.


*Clinical & Developmental Immunology*, March 2005; 12(1): 85–90

Taylor & Francis

Taylor & Francis Group

**PRELIMINARY REPORT**

### A novel system to diagnose cutaneous adverse drug reactions employing the cellscan—comparison with histamine releasing test and Inf-γ Releasing Test

ILAN GOLDBERG[1], BORIS GILBURD[2], MARTINE SZYPER KRAVITZ[2], SHMUEL KIVITY[3], BERTA BEN CHAIM[4], TIRZA KLEIN[4], YAEL SCHIFFENBAUER, ELA TRUBNIYKOVR, SARAH BRENNER[1], & YEHUDA SHOENFELD[2,5]

[1]*Department of Dermatology, Tel Aviv-Sourasky Medical Center, Tel Aviv, Israel,* [2]*Department of Medicine B, Center for Autoimmune Diseases, Sheba Tel-Hashomer, Israel,* [3]*The Chest and Allergy Institute, Tel Aviv-Sourasky Medical Center, Tel Aviv, Israel,* [4]*Drug hypersensitivity laboratory and Tissue Typing Laboratory, Rabin Medical Center, Beilinson Campus, Tel Aviv, Israel, and* [5]*Incumbent of the Laura Schwatz-Kipp Chair for Research on Autoimmune Diseases, Tel Aviv University, Tel Aviv, Israel*

**Abstract**

*Background*: There are several mechanisms to describe allergic drug reactions yet the methods to diagnose them are limited.

*Objective*: To compare several conventional clinical and laboratory methods to diagnose skin reactions to drugs to a new method of diagnosing drug reactions by the CellScan system.

*Methods*: The study entailed 21 patients who were diagnosed as suffering from drug eruptions, and 105 healthy controls with no history of drug allergy. The drugs were classified into two groups according to suspicion of causing drug allergy: high and low. Most of the patients were on more than one drug, leading to 41 patient–drug interactions (assays). Histamine releasing test (HRT), interferon (INF)-γ releasing test and CellScan examination were performed on lymphocytes of the patients and controls.

*Results*: The HRT was interpreted as positive in 9 out of 18 (50%) patients and in 13 out of 35 (37%) assays. Based on the INF-γ releasing test, positive results were observed in 16 out of 21 (76%) patients and in 24 out of 41 (59%) assays. In the CellScan test (CST), positive results were observed in 17 out of 21 (81%) patients and in 29 out of 41 (71%) assays. The rate of identifying the drug for eruption in the high suspicion level drugs was 9 out of 22 (41%) assays in the HRT, 20 out of 24 (83%) assays in the INF-γ releasing test, and 21 out of 24 (87%) studies with the CellScan method. The rate of determining of the drug that caused the eruption in the low suspicion level drugs was 4 out of 13 (31%) in the HRT, 4 out of 17 (24%) assays in the INF-γ releasing test, and 8 out of 17 (47%) analyses in the CST. When examined in the CellScan, 99 out of 105 (94%) controls were interpreted as negative.

*Conclusion*: This preliminary study indicates that the CellScan seems to be an easy and promising method for the detection of drugs responsible for adverse skin reactions. In contrast to the HRT and to the Interferon-γ secretion test, the CellScan method is characterized by its ability to track and monitor the reaction of individual cells. By measuring the kinetic parameters of selected cells before and after adding the suspected drug, we were able to identify the culprit drug. The CellScan method had the highest sensitivity, and the interferon-γ secretion test had the highest specificity for detection of the culprit drug. In contrast, the analysis of 105 normal control sera disclosed a high specificity of 94% for the CellScan method.

**Keywords:** *Drug allergy, cytokines, T cells, histamine, interferon releasing test*

### Introduction

Adverse reactions to drugs or biological agents are frequent consequences of medical therapy, since few if any medications are free of adverse effects. Allergic and other immunological drug reactions (type B reactions), cause up to 24% of the observed adverse drug reactions, in a primarily in-patient population (Lazarou et al. 1998). For most drugs, the risk of an allergic reaction is 1–3 percent (DeSwarte et al. 1993). Between 2 and 24% (mean 11%) of hospitalized patients experience adverse drug reactions (ADR), and

Correspondence: I. Goldberg, Address: Department of Medicine B, Center for Autoimmune Diseases, Chaim Sheba Medical Center, Tel-Hashomer 52621, Israel. Tel: 972 3 5302652. Fax: 972 3 5352855. E-mail: shoenfel@post.tau.ac.il

ISSN 1740-2522 print/ISSN 1740-2530 online © 2005 Taylor & Francis Group Ltd.
DOI: 10.1080/10446670410001722230

the incidence of serious ADR requiring hospitalization is 4.7% (Lazarou et al. 1998). Fatalities occur in 1 of 10,000 allergic drug reactions (Boston Collaborative Drug Surveillance Program 1973), and they are reported in 0.01% of the surgical inpatients and in 0.1% of the medical inpatients (Armstrong et al. 1976, Porter and Jick 1977). A meta-analysis of 39 prospective studies on the incidence of ADR in hospitalized patients over a period of 30 years concluded that fatal reactions ranked between the fourth and sixth leading cause of death in the United States (Lazarou et al. 1998).

Cutaneous reactions are the most common manifestation of drug reactions, and include maculopapular eruptions, urticaria, angioedema, bullous exfoliative rashes, photosensitivity reactions, fixed drug eruptions and leucocytoclastic vasculitis (Shefferd 2003).

The clinical diagnosis of drug-induced skin reactions is not an easy task. On the one hand the allergic reaction may present in different and variable skin manifestations. On the other hand, skin reactions may mimic non-allergic skin diseases (Halevy and Feuerman 1984, Brenner et al. 1998). Other confounding factors inthe task of diagnosing drug-induced skin reactions are the frequent polypharmacy patients are exposed to, and the latent period that can last from days to months, between taking the drug and the appearance of the skin reaction.

To date reliable tests that confirm allergic reaction exist only for selected drugs (e.g. standardized skin test for penicillin and local anesthetics, specific IgE, patch test). In the vast majority of drug reactions, diagnosis is based on clinical parameters and lacks objective confirmation. Challenge tests are potentially hazardous and in most clinical situations are not recommended. Due to these limitations several *in vitro* tests have been developed, with the intent to elucidate the different mechanisms of drug reactions, and to provide evidence for the association between a suspected drug and the adverse reaction (Wide and Juhlin 1971, Gimenez-Camarasa et al. 1975, Watson et al. 1978, Shoenfeld et al. 1980, Shoenfeld 1982, Grunwald et al. 1989, Halevy et al. 1991, Halevy et al. 2001, Sachs et al. 2001, Goldberg et al. 2004). The value of these *in vitro* tests are also limited in that the parent drug is used, and reactions occurring to drugs metabolites or to the drug associated with carrier proteins, are missed.

To date no objective method exists with enough sensitivity and specificity to objectively diagnose drug reactions, and there is no gold standard test to compare the results of different assays.

The aim of this study was to compare conventional clinical and laboratory methods used for the diagnosis of drug-induced skin reactions, such as the histamine releasing test (HRT) and to the Interferon-γ secretion test, and to introduce a novel method of diagnosing drug reactions employing the CellScan system. In contrast to the HRT and to the Interferon-γ secretion test, the CellScan method enables tracking and monitoring of the reaction of individual cells. As such, analysis can be performed to characterize individual cell behavior, and its kinetics under different conditions or drug exposure.

### Patients and methods

### *Patients*

Patients were selected after they presented with skin reactions compatible with drug eruptions, after exposure to different drugs. The suspected drugs were classified into two groups, high and low level of suspicion, based upon established parameters in drug-allergy clinical practice including: (1) the prevalence of allergic skin reactions for the specific drug; (2) the temporal relationship between drug exposure and the development of the skin rash; (3) the exclusion of other medical conditions or drugs that can mimic the specific skin reaction (Adkinson 2003). Most of the patients were exposed to more than one drug, and for every drug a separate evaluation was performed.

Sera from 105 healthy asymptomatic adults were used as negative controls for the CellScan test (CST).

### *Histamine releasing test*

The HRT is based on the measurement of histamine released from guinea pig mast cells after incubation with the patient's serum and the suspected drug. Briefly, venous blood was collected from the patients. Guinea pig mast cells were incubated for 60 min. with the patient's serum and guinea pig's serum as a control. Next the suspected drug was added for an additional 15 min. of incubation. Following the incubation, the supernatants were collected for the detection of histamine secretion by the ELISA technique (Immunotech, Westbrook ME, USA).† The test was interpreted as the number of degranulated cells in the presence of the drug, compared to the cells in the absence of the drug (the difference in percentage). Negative result was determined when the value was between 0 and 20%, borderline: 21–27%, weak positive: 28–30%, positive: over 31%.

### *In Vitro interferon-γ release Test (IRT)*

The technique of *in vitro* INF-γ releasing test was used according to the method published by Halevy *et al.* (1998). Briefly, the patient's lymphocytes were cultured for 24 h in a medium containing PHA-P, 5%

†ELISA tech for histamine.

fetal calf serum with or without the tested drug (unmodified and dissolved in the appropriate solvent). Following incubation, the supernatants were collected for the detection of IFN-γ using a comercial ELISA kit (Quantikine kit, R & D Systems, Minneapolis,USA). For each drug the increase in IFN-γ release (in percentage) was calculated.

### *CellScan test*

The CellScan apparatus is a laser scanning cytometer incorporating a unique cell carrier that allows repeated, high-precision fluorescence intensity (FI) and fluorescence polarization (FP) measurements to be made on intact living cells under physiological conditions (Deutsch and Weinreb 1994, Kaplan et al. 1997). The CellScan is unique in its capability of repetitive measurements of individual cells in a cell population. It has been used to detect activation of lymphocytes both in physiological (Eisenthal et al. 1996, Zurgil et al. 1996) and pathological situations including: cancer (Ron et al. 1995, Merimsky et al. 1996, Rahmani et al. 1996, Schiffenbauer et al. 2002, Cohen et al. 2003), autoimmune disorders (Zurgil et al. 1997, Zurgil et al. 1999), and atherosclerosis (Zurgil et al. 1999).

*Loading cells onto the cellcarrier.* The cellcarrier was placed on a specially designed loader. By tightening the screws of the cell loader on the silicon plugs, a small positive pressure in the buffer chamber was created. Most of the drop that was formed on the top of the cellcarrier was removed by suction. After a drop of the cell suspension was added to the cellcarrier, the screws were gradually released, and cells were siphoned into the traps. The remaining cell suspension was removed, and replaced with fresh buffer.

*Measurement of fluorescence polarization changes by cellScan induced by different drugs stimulus.* The polarization of a molecule is proportional to the molecule's rotational relaxation time, which is related to viscosity ($\eta$), absolute temperature ($T$), molecular volume ($V$), and the gas constant ($R$). Rotational relaxation time $= 3\eta V/RT$. Therefore, at a given temperature, changes in viscosity or molecular volume as a result of degradation, binding, dissociation or conformational changes will be reflected in the polarization value. Specifically, after incubation with the appropriate antigen, the cells were stained with fluorescein diacetate (FDA), which is a lipophilic, uncharged, and a non-fluorescent esther that can readily cross cell membranes. Once inside the cell, it is hydrolyzed by non-specific esterases to produce the fluorescein anion, which is retained by living cells and is lost by cells with damaged membranes. Changes in viscosity of the cytoplasm as a result of cell activation lead to (FDA) depolarization.

The cells from the patient's and from the control's groups were incubated with or without the corresponding drugs for 30 min at 37°C in a humidified atmosphere containing 5% CO2. Next, they were stained for 5 min with FDA at a final concentration of 2.4 μM, loaded onto the Cellcarrier, washed twice with PBS, and FP was measured. Reduction in intracellular FP of cells after antigenic stimuli in comparison to unstimulated cells reflect the extension of cell activation. Results of CellScan examination were expressed as mean % of stimulation ± standard deviation (Mean ± SD).

### Results

Twenty-one patients, 12 women and 9 men, with an average age of 65 were recruited to this study. The most common allergic skin presentation associated with drug exposure was maculopapular rash (11 patients), followed by urticaria and/or angioedema (8 patients). Two patients presented with bullous rashes. None of the patients were hospitalized and all the patients were followed in our outpatient clinic until full resolution of the adverse reactions. Median time to full recovery was ten days. There were no fatalities among our patients.

Twenty-one patients were evaluated for 41 suspected drug reactions, and Table I summarizes the demographic data of the study population. Eight patients were exposed to one drug only, and only one drug was analyzed. Thirteen patients were exposed to multiple drugs: seven patients to two drugs, five patients to three drugs and in one patient four possible drugs were evaluated (Table II). Among the 21 patients with suspected drug-induced skin reactions, in 18 patients, the three different assays were used, and in the remaining three patients due to technical reasons only INF-γ release test and Cellscan were compared.

For each test we calculated the total number of patients with positive results, the total number of assays that were positive (Table III), and the number of positive results for each assay in relation to the clinical suspicion (Table IV).

### *Histamine releasing test*

Positive results were observed in 9 out of 18 (50%) patients and in 13 out of 35 (37%) assays. In drugs with high clinical suspicion 9 out of 22 (41%) assays were positive, and 2 out of 13 (31%) assays were positive in low clinical suspicion drugs.

### *INF-γ releasing test*

The test was positive in 16 out of 21 (76%) patients, and in 24 out of 41 (59%) assays. When results were stratified according to high or low clinical suspicion 20 out of 24 high clinical suspicion drugs gave positive results (83%), compared to 4 positive results among 17 low clinical suspicion drugs (24%).

Table I. Patients' demographics, skin reactions, drug exposure and tests results.

| No. | Age | Sex | Reaction* | Drug | IFN γ | Histamine | CellScan | Suspicion |
|---|---|---|---|---|---|---|---|---|
| 1 | 62 | F | Mac pap | Coumarin | P | P | N | H |
| 2 | 48 | F | Mac pap | Cefuroxime | P | P | P | H |
| | | | | Roxythrocine | P | P | P | H |
| | | | | Ofloxacin | P | P | N | L |
| 3 | 57 | M | Mac pap | Atenolol | P | N | P | H |
| | | | | Sod.valproate | N | N | N | L |
| 4 | 82 | M | Mac pap | Phenytoin | N | N | P | H |
| 5 | 76 | M | Mac pap | Simvastatin | N | N | N | L |
| | | | | Cilazapril | P | N | P | H |
| | | | | Aspirin | N | N | P | L |
| 6 | 70 | M | Mac pap | Rofecoxib | P | N | P | H |
| 7 | 45 | F | Bullous | Benazepril | P | – | P | H |
| | | | | Metformin | P | – | N | L |
| | | | | Timolol | N | – | P | L |
| 8 | 64 | F | Urticaria | Amoxycillin | N | N | P | H |
| | | | | Paracetamol | P | P | N | H |
| | | | | Famotidine | P | P | P | H |
| 9 | 82 | F | Mac pap | Cilazapril | P | P | P | H |
| | | | | Simvastatin | P | N | P | H |
| | | | | Paracetamol | N | P | P | L |
| | | | | Nifedipine | N | N | N | L |
| 10 | 83 | F | Urticaria | Pseudoephedrine | P | P | N | H |
| 11 | 24 | M | Rash | Pergonal | N | N | P | L |
| | | | | Bromocriptine | P | N | P | H |
| 12 | 70 | F | Rash | Fosinopril | N | P | P | H |
| 13 | 67 | M | Rash | Aspirin | P | N | P | H |
| | | | | Aspirin | P | N | P | H |
| 14 | 46 | F | Rash | Phenazopyridine | N | N | P | L |
| | | | | Ibuprofen | P | N | P | H |
| | | | | Cefuroxime | N | P | N | L |
| 15 | 79 | F | Rash | Enalapril | N | N | P | L |
| 16 | 75 | F | Rash | Simvastatin | N | N | P | L |
| | | | | Ramipril | N | P | P | H |
| 17 | 88 | M | Rash | Indometacin | P | P | N | L |
| 18 | 68 | M | Rash | Penicillin | P | N | P | H |
| | | | | Erythromycin | P | N | P | H |
| 19 | 70 | M | | Simvastatin | P | N | P | H |
| | | | | Enalapril | N | N | P | L |
| 20 | 55 | F | AGEP | Acetaminophen | P | NE | P | H |
| | | | | Dipyrone | P | NE | N | L |
| 21 | 59 | F | Bullous | Colchicines | N | NE | N | L |

P–positive, N–negative, H–high clinical suspicion, L–low clinical suspicion.
* Mac Pap–Maculopapaular rash, AGEP–acute generalized exanthematous pustulosis.

*CellScan test*

Using the CellScan technique 17 out of 21 (81%) patients had positive results, and 29 out of 41 (71%) assays were interpreted as positive. These results increased for high suspicion drugs where 21 out of 24 (87%) of assays were positive. For the low-suspicion drugs 8 out of 17 (47%) gave positive results.

The rate of identification of the drug that caused the eruption in the high suspicion level drugs was 9 out of 22 (41%) assays in the histamine release test, 20 out of 24 (83%) assays in the interferon release test, and 21 out of 24 (87%) assays in the CST (Table III). In the low suspicion level drugs, the rate of identification of the drug that caused the eruption was 4 out of 13 (31%) assays in the histamine release test, 4 out of 17 (24%) assays in the interferon release test, and 8 out of 17 (47%) assays in the CST (Table IV).

Table II. Drug exposure by number of patients who developed adverse drug reactions.

| Number of patients | Number of drugs |
|---|---|
| 8 | 1 |
| 7 | 2 |
| 5 | 3 |
| 1 | 4 |

*Controls*

Ninety-nine out of 105 (94%) controls were negative using the CST.

Table III. Number (%) of patients with adverse reactions and positive results in the different tests.

| | Number (%) of patients with positive result* | Number (%)of assays with positive result* |
|---|---|---|
| Histamine release | 9/18 (50%) | 15/35 (37%) |
| Interferon-γ release | 16/21 (76%) | 24/41 (59%) |
| CellScan | 17/21 (81%) | 29/41 (71%) |

* Positive results in each test as defined in the methods section.

Table IV. Positive and negative results in assays stratified according to clinical suspicion among the different tests.

| | High suspicion* | | Low suspicion* | |
|---|---|---|---|---|
| | +Assay | − Assay | +Assay | − Assay |
| Histamine release | 9/22 (41%) | 13/22 (59%) | 4/13 (31%) | 9/13 (69%) |
| Interferon- γ release | 20/24 (83%) | 4/24 (17%) | 4/17 (24%) | 13/17 (76%) |
| CellScan | 21/24 (87%) | 3/24 (13%) | 8/17 (47%) | 9/17 (53%) |

* The suspected drugs were classified into high and low level of suspicion based upon: the prevalence of allergic skin reactions for the specific drug; the temporal relationship between drug exposure and the development of the skin rash; and the exclusion of other medical conditions or drugs that can mimic the specific skin reaction.

### Discussion

Adverse reactions to pharmaceutical and diagnostic products constitute a major hazard in the practice of medicine and are responsible for substantial morbidity and cost (Adkinson 2003). Currently, there are no highly specific tests that are predictive either of the capacity of novel compounds (drugs) to induce allergic reactions, or of the susceptibility of individuals to experience allergic reaction. *In vitro* cytokine release may have a diagnostic role in cutaneous drug eruptions. In delayed type hypersensitivity reactions (type IV) Th1 lymphocytes are activated and produce IL-2 and interferon-γ, whereas in immediate (type I) hypersensitivity reactions Th2 lymphocytes are activated and produce IL-4, IL-5 and IL-10, which are responsible for specific IgE production. Recent studies have demonstrated the diagnostic potential of a new test based on release of interferon-γ from lymphocytes after exposure to a suspected drug with a sensitivity of 54% and specificity of 92% (Halevy et al. 2001).

In the present study we compared the HRT and the interferon-γ secretion test to a novel cell-based technology, for the diagnosis of suspected drug-induced skin reactions. Analysis of the performance of the three tests in detecting drugs responsible for skin reactions, based on the clinical suspicion disclosed a low sensitivity (41%) and specificity (69%) for the HRT, a relative high sensitivity (83%) and moderate specificity (76%) for the Interferon-γ secretion test, and a high sensitivity (87%) and moderate-low specificity (53%) for the CellScan (see Table IV). The CellScan method had the highest sensitivity, and the interferon-γ secretion test had the highest specificity for detection of the culprit drug. In contrast, the analysis of 105 normal control sera disclosed a high specificity of 94% for the CellScan method.

In contrast to the HRT and to the Interferon-γ secretion test, the CellScan method is characterized by its ability to track and monitor the reaction of individual cells. As such, analysis can be performed to characterize individual cell behavior, thus establishing its kinetic trends. Kinetic approaches are valuable tools for biological studies of cell function. By measuring the kinetic parameters of selected cells before and after adding the suspected drug, we were able to identify the culprit drug.

It is clear that the future direction of predictive drug allergy testing is not with animal tests (that are a poor indicator of human responses) but with the use of *in vitro* human cell culture systems that model the human *in vivo* immune response. Our preliminary study indicates that the CellScan seems to be a promising method for the detection of drugs responsible for adverse skin reactions.

### References

Adkinson NK. 2003. Drug allergy. In: Adkinson NK, Yunginger JW, Busse WW, Bochner BS, Holgate ST, Simmons ER, editors. Middleton's allergy: Principles and practice. 6th ed. Mosby. p 1679–1694.

Armstrong B, Dinan B, Jick H. 1976. Fatal drug reactions in patients admitted to surgical services. Am J Surg 132:643–645.

Boston Collaborative Drug Surveillance Program 1973. Drug-induced anaphylaxis: A cooperative study. JAMA 224:613–615.

Brenner S, Bialy-Golan A, Ruocco V. 1998. Drug-induced pemphigus. Clin Dermatol 16:393–397.

Cohen CJ, Denkberg G, Schiffenbauer YS, Segal D, Trubniykov E, Berke G, Reiter Y. 2003. Simultaneous monitoring of binding to and activation of tumor-specific T lymphocytes by peptide-MHC. J Immunol Methods 9346:1–14.

DeSwarte RD. 1993. Drug.allergy. In: Patterson R, Grammer LC, Greenberg PA, Zeiss CR, editors. Allergic diseases: Diagnosis and management. 4th ed. Philadelphia, PA: JB Lippincott. p 395–551.

Deutsch M, Weinreb A. 1994. Apparatus of high precision repetitive sequential optical measurement of living cells. Cytometry 16:214–226.

Eisenthal A, Marder O, Dotan D, Baron S, Lifschitz-Mercer B, Chaitchik S, Tirosh R, Weinreb A, Deutsch M. 1996. Decrease of intracellular fluorescein fluorescence polarization (IFFP) in human peripheral blood lymphocytes undergoing stimulation with phytohaemagglutinin (PHA), concanavalin A (ConA), pokeweed mitogen (PWM) and anti-CD3 antibody. Biol Cell 86:145–150.

Gimenez-Camarasa JM, Garcia-Calderon P, De Moragas JM. 1975. Lymphocyte transformtion test in fixed drug eruption. N Engl J Med 292:819–821.

Goldberg I, Gilburd B, Shovman O, Brenner S. 2004. Clinical and laboratory assays in the diagnosis of cutaneous adverse drug reactions. Isr Med Assoc J 6:50–51.

Grunwald MH, Halevy S, Livni E. 1989. Allergic vasculitis induced by hydrochlorthiazide: Confirmation by mast cell degranulation test. Isr J Med Sci 25:572–574.

Halevy S. 1998. *In-vitro* tests for adverse drug reactions based on cytokine release. In: Kauppinen K, Alanko K, Hannuksela M, Maibach H, editors. *In-vitro* tests for adverse drug reactions based on cytokine release. Boca Raton, FL: CRC Press LLC. p 119–131.

Halevy S, Feuerman EJ. 1984. (Heb) drug induced eruptions which mimic skin diseases. Fam Physician 12:267.

Halevy S, Grunwald MH, Sandbank M, et al. 1991. Macrophage migration inhibition factor (MIF) as a diagnostic aid in drug eruptions. Harefuah 121:147–149.

Halevy S, Cohen AD, Grossman N. 2001. *In vitro* interferon gamma release in diagnosis of cutaneous adverse drug reactions. Harefuah 140:121–124.

Kaplan MR, Trubniykov E, Berke G. 1997. Fluorescence depolarization as an early measure of lymphocyte stimulation. J Immunol Methods 201:15–24.

Lazarou J, Pomeranz BH, Corey PN. 1998. Incidence of adverse drug reactions in hospitalized patients. A meta-analysis of prospective studies. JAMA 279:1200–1205.

Merimsky O, Deutsch M, Tirosh R, Wohl I, Weinreb A, Chaitchik S. 1996. Detection of colon cancer by monitoring the intracellular fluorescein fluorescence polarization changes in lymphocytes. Cancer Detection and Prevention 20:300–307.

Porter J, Jick H. 1977. Drug-related deaths among medical inpatients. JAMA 237:879–881.

Rahmani H, Deutsch M, Ron I, Gerbat S, Tirosh R, Weinreb A, Chaitchik S, Lalchuk S. 1996. Adaptation of the Cellscan technique for the SCM test in breast cancer. Eur J Cancer 32A:1758–1765.

Ron IG, Detusch M, Tirosh R, Weinreb A, Eisenthal A, Chaitchik S. 1995. Fluorescence polarization changes in the lymphocytic cytoplasm in the various stages of brest cancer. Eur J Cancer 6:917–920.

Sachs B, Ederman S, Al-Masaoudi T, Merk HF. 2001. *In vitro* drug allergy detection system incorporating human liver microsomes in chlorazepate-induced skin rash: Drug-specific proliferation associated with interleukin-5 secretion. Br J Dermatol 144:316–320.

Schiffenbauer YS, Trubniykov E, Zacharia BT, Gerbat S, Rehavi Z, Berke G, Chaitchik S. 2002. Tumor sensitivity to anti-cancer drugs predicted by changes in fluorescence intensity and polarization *in vitro*. Anticancer Res 22:2663–2670.

Shefferd GM. 2003. Hypersensitivity reactions to drugs: Evaluation and management. Mt Sinai J Med 70:113–125.

Shoenfeld Y. 1982. "Leukopenia: Idiopathic or drug-induced"—how to differentiate? N Engl J Med 307:251.

Shoenfeld Y, Livni E, Shaklai M, Pinkhas J. 1980. Sensitization to ibuprofen in systemic lupus erythematosus. JAMA 244:547–548.

Watson B, Rhodes EL, Rosling AE. 1978. The release of beta glucuronidase from the white cells of patients with drug rashes when incubated in autologous plasma, with and without the addition of the causal drug. Br J Dermatol 99:183–190.

Wide L, Juhlin L. 1971. Detection of penicillin allergy of the immediate type by radioimmunoassay of reagins (IgE) to penicilloyl conjugates. Clin Allergy 1:171.

Zurgil N, Deutsch M, Tirosh R, Brodie C. 1996. Indication that intracellular fluorescence polarization of T lymphocytes is cell cycle dependent. Cell Struct Funct 21:271–276.

Zurgil N, Gerbat S, Langevitzh P, Tishler M, Ehrenfeld M, Shoenfeld Y. 1997. Intracellular fluorescence polarization measurements by the cellscan system: Deteaction of cellular activity in autoimmune disorders. Isr J Med Sci 33:273–279.

Zurgil N, Gerbat S, Langevits P, Tishler M, Ehrenfeld M, Kaufman M, Deutsch M, Shoenfeld Y. 1999. Detection of cellular activity in autoimmune disorders by the cellscan system. In: Shoenfeld Y, editor. The decade of autoimmunity. V. Elsevier Science, B.V. p 295–304.

Zurgil N, Levy M, Deutsch M, Gilburd B, George J, Haratz D, Kaufman M, Shoenfeld Y. 1999. Reactivity of peripheral blood lymphocytes to oxidized low-density lipoprotein: A novel system to estimate atherosclerosis employing the cellscan. Clin Cardiol 22:526–532.

